# A Combination of Outcome and Process Feedback Enhances Performance in Simulations of Child Sexual Abuse Interviews Using Avatars

**DOI:** 10.3389/fpsyg.2017.01474

**Published:** 2017-09-11

**Authors:** Francesco Pompedda, Jan Antfolk, Angelo Zappalà, Pekka Santtila

**Affiliations:** ^1^Faculty of Arts, Psychology and Theology, Åbo Akademi University Åbo, Finland; ^2^Turku Brain and Mind Center Turku, Finland; ^3^CRIMELAB, Istituto Universitario Salesiano Torino Rebaudengo, Salesian Pontifical University Turin, Italy; ^4^Faculty of Arts and Sciences, NYU Shanghai Shanghai, China

**Keywords:** child sexual abuse, investigative interviewing, serious gaming, interview training, feedback

## Abstract

Simulated interviews in alleged child sexual abuse (CSA) cases with computer-generated avatars paired with feedback improve interview quality. In the current study, we aimed to understand better the effect of different types of feedback in this context. Feedback was divided into feedback regarding conclusions about what happened to the avatar (outcome feedback) and feedback regarding the appropriateness of question-types used by the interviewer (process feedback). Forty-eight participants each interviewed four different avatars. Participants were divided into four groups (no feedback, outcome feedback, process feedback, and a combination of both feedback types). Compared to the control group, interview quality was generally improved in all the feedback groups on all outcome variables included. Combined feedback produced the strongest effect on increasing recommended questions and correct conclusions. For relevant and neutral details elicited by the interviewers, no statistically significant differences were found between feedback types. For wrong details, the combination of feedback produced the strongest effect, but this did not differ from the other two feedback groups. Nevertheless, process feedback produced a better result compared to outcome feedback. The present study replicated previous findings regarding the effect of feedback in improving interview quality, and provided new knowledge on feedback characteristics that maximize training effects. A combination of process and outcome feedback showed the strongest effect in enhancing training in simulated CSA interviews. Further research is, however, needed.

## Introduction

The poor quality of investigative interviews in alleged child sexual abuse (CSA) cases is a worldwide problem, as highlighted by several international studies in different countries (e.g., Cederborg et al., 2000; Johnson et al., 2015). This constitutes a major problem as in a majority of cases the interview with the child is the only available evidence (e.g., Lamb et al., 2008). To face the current problem with poor interview quality, several different training programs have been developed. Due to the difficulty in developing training programs that provide training in a realistic context, but where mistakes are not very costly, so-called serous gaming paradigms have recently been employed in the context of interview training (e.g., Brubacher et al., 2015; Powell et al., 2016). Serious gaming has been successful in changing and maintaining expert behavior in different contexts, in a time-and-cost effective manner (Wouters et al., 2013; van Dijk et al., 2015).

When interviewing children, the use of open-ended questions (Orbach and Lamb, 2000) and the avoidance of closed questions (e.g., Lamb et al., 1998) is of vital importance. Research shows that theoretical knowledge of interview principles alone is not a reliable way to improve the quality of CSA interviews. A study looking at professionals in England and Wales who were trained to follow the memorandum of good practice (Sternberg et al., 2001) provides an example for the previous statement. Despite undergoing training, interview quality remained similar in England and Wales and comparable to the quality in countries in which these guidelines had not been implemented.

A possible explanation for the poor quality of interviews relates to how training and feedback is delivered (Benson and Powell, 2015). To be effective, training must be associated with feedback. Feedback must, in turn, be immediate, detailed (Smith, 2008) and continuous (Lamb et al., 2002). For example, Price and Roberts (2011) showed that intensive training accompanied by continuous process feedback improved interview quality in CSA interviews.

As suggested by Hattie and Timperley (2007), even if feedback is a powerful resource, the effects on training are influenced by the type of feedback provided. The results of a vast meta-analysis presented in Hattie and Timperley (2007) showed how process feedback, which focus on the task processes’ and provide info on how to perform the task (Landsberg et al., 2010), is more effective when the learning goal is the transfer of acquired skills to more complex tasks. While outcome feedback, which can be defined as the feedback “about how a task is being accomplished or performed” (Hattie and Timperley, 2007, p. 91), is more efficient in changing faulty interpretations.

However, the literature shows consensus about a general stronger effect of process feedback on training of complex tasks (e.g., Astwood et al., 2008). In addition, a more recent meta-analysis of feedback in a computerized environment (Van der Kleij et al., 2015) showed that elaborate feedback (process feedback) produced larger effect sizes compared to other types of feedback based on the correctness of the response (outcome feedback).

In an investigative interview context, process feedback can be construed as feedback on whether the questions used by the interviewer are appropriate or not. Instead, feedback on whether the interviewer reached the correct conclusion about what had happened to the avatar corresponds to outcome feedback. In an earlier study (Pompedda et al., 2015), participants were provided with both types of feedback simultaneously in simulated investigative interviews. The simultaneous use of a combination of process and outcome feedback (vs no feedback) improved the quality of the simulated investigative interviews. Because both feedback types were administered simultaneously in this study, the researchers were unable to exhaustively investigate how the two types of feedback influenced the learning process.

The effects of process feedback on question types and or behavior employed by the interviewer has been previously investigated in CSA investigative interviews’ (e.g., Lamb et al., 2002; Benson and Powell, 2015). To our best knowledge, however, no study within the field of CSA investigative interviews has tested the effects of a combination of process and outcome feedback in comparison to these two type of feedback provided separately. As highlighted by Hershkowitz et al. (2017), the common feedback provided to interviewers in training programs pertains to the interviewer’s behavior, for example, feedback on the question types used (e.g., Benson and Powell, 2015; Yi et al., 2016). Outcome feedback can be provided at the end of quizzes regarding best practice (e.g., Powell et al., 2016), but rarely to the conclusion of the interviewer. A possible explanation for this gap is that knowing the ground truth of CSA cases in a real context is rarely possible.

In the present study, we used simulated interviews with computer-generated avatars to test the effects of the two types of feedback, separately and in combination, on different variables measuring improvements of interview quality. Differently from real cases, within this setup it is always possible to know the ground truth of the story and thereafter to provide detailed feedback.

In line with previous literature, we expected that outcome feedback alone would have the weakest effect, as it only provides information on mistakes. We also expected that process feedback would have a stronger effect compared to outcome feedback as it provides information on how to change. Finally, we expected that the combination of both types of feedback would be the most effective because it provides both a reason for change and information on how to change.

The participants took part in a simulation of an alleged CSA case. First, we provided the participants with a short scenario describing the child (e.g., age, family composition) and the allegation of abuse. In the simulated interview environment, the avatars possessed predefined memories (half of the avatars possessed memories of abuse, half of them did not). The revelations of these memories were linked to the question-types used by the interviewer via a series of response algorithms.

In the present study a good interview was defined by (a) a higher proportion of recommended questions out of all questions, (b) a higher number of relevant and neutral details and a lower number of wrong details found out by the interviewer, and (c) a higher percentage of correct conclusions reached.

We formulated the following hypotheses regarding the effects of feedback:

Hypothesis 1: The participants receiving feedback will conduct better interviews compared to the control group.
Hypothesis 2: The group receiving process feedback will conduct a better interview compared to the group receiving outcome feedback.
Hypothesis 3: The group receiving both types of feedback simultaneously will conduct a better interview compared to participants who received only one of the two types of feedback.

## Materials and Methods

### Participants

The sample consisted of 48 participants (10 men, *M* = 28 years, *SD* = 9), recruited from two different psychology departments in Italy, and randomly assigned to four different conditions. Of the participants, seven were graduate students in psychology, and 41 were undergraduate students in psychology. One-way ANOVA did not show differences between groups for age, *F*(3,44) = 1.00, *p* = 0.404. The Levene statistic for homogeneity of variance was significant; however, a subsequent Brown–Forsythe robust test was not significant (*p* = 0.41). A Fisher–Freeman–Halton’s test on a 4 (Groups) × 2 (Gender) contingency table showed no difference for gender (*p* = 1.0). We evaluated the university degree as ordinal variable, with 1 as lowest degree (no degree), 2 as bachelor degree, and 3 as master degree; a Kruskal–Wallis chi-squared did not show differences for what concerns the acquired university degree [*H*(3,48) = 7.198, *p* = 0.066]. The data collection of this study is part of a larger project for which ethical permission has been granted from the Ethics Board of the Department of Psychology and Logopedics at Åbo Akademi. Other results from the same data collection have been published in Pompedda et al. (2015).

### Designs

The study used a between subjects-design with four different conditions, each corresponding to a separate experiment group. The first group received no feedback; the second group received outcome feedback; the third group received process feedback; and the fourth group received both types of feedback simultaneously. The interviewers performed four interviews. For these interviews, four different avatars were selected to account for all the possible combinations of age, gender, and abuse or not-abuse. Participants thus interviewed two abused avatars and two not abused avatars balanced for age and sex. The order of these interviews was randomized. For their participation, participants received a movie ticket and were able to leave the experiment at any moment if they felt uncomfortable.

### Materials

#### Simulations of Investigative Interviews

The Empowering Interviewer Training (EIT) software consisted of eight different avatars, two 4-year-old male, two 4-year-old female, two 6-year-old male, and two 6-year-old female avatars. Each avatar contained memories of different scenarios of alleged CSA. For half of the avatars, the scenario contained memories of sexual abuse, for the other half it did not. The avatars also expressed variations in emotionality, some avatars showed emotions, such as facial expression and crying, whereas others did not. We created two different response algorithms, one for the 4-year-old and one for the 6-year-old avatars. The algorithms are based on the best available empirical knowledge about children’s memory and suggestibility (Pompedda et al., 2015). The use of algorithms thus allows for a realistic simulation of how real children would respond. For example, if an interviewer asks a multiple choice question regarding a detail that is not present in the memory of a child, the child sometimes chooses one of the options even if none of them corresponds to the child’s memory of the event. The interviewer might ask “Was your dad or your uncle at home?” to which the child might respond “My uncle,” although this is untrue. In this way, the interviewer can create wrong details.

For each scenario, we created lists of details that constituted the memories the avatar remembered. In this way it was possible for us to objectively define whether the interviewers correctly found out what had happened to the avatar. We divided the details present in the avatars’ memory in:

(1) Relevant details: These details were present in the avatar memory and related to the allegation. If the avatar had been abused, relevant details represented the description of the abuse. Otherwise, they represented an innocent explanation for the allegation.
(2) Neutral details: These details were present in the avatar memory but not related to the alleged abuse situation. For example, they contain information about people or the avatar’s favorite games.

The predefined details allowed us to evaluate better the interview, to provide detailed feedback and to recognize the wrong details, which were details not present in the predefined memory but created by the interviewer using not-recommended questions.

The avatars’ images were created morphing different images of real children, subsequently animated using the software (SitePal, 2014) to create a series of video clips containing all the predefined answers of the child.

### Procedures

The participants arrived into the EIT laboratory. Upon arrival, a research assistant provided participants with a paper explaining the aim of the study and the task, after which the participant signed an informed consent form. Before each interview, each participant was provided with a paper containing a description of the abuse allegation and some personal information regarding the “child.” Participants sat in front of a computer monitor where the videos of the avatars were displayed. When the interview started, the participant verbally asked the questions facing the monitor and based on the question they used, an operator who sat in another room, launched the appropriated video-clip based on the algorithms. For example, after two open-ended questions the operator launched a video with the first detail regarding the avatar story. Participants were informed of the possibility to conclude the interview whenever they preferred, but within a maximum of 10 min. Participants were also instructed to conduct the interview in the way they thought was most appropriate and to focus on the investigation of the alleged abuse situation. At the end of the interview, each participant provided a dichotomous decision regarding the alleged sexual abuse and provided as much details as they could regarding what had happened. For example, in an abuse scenario it was mandatory to provide information regarding who was the abuser and how the abuse had taken place. In order to classify a conclusion as correct, all the information regarding the scenario had to be correct. At the end of the training, we provided the participants with a questionnaire in which they were given a chance to express their feelings. There were no cases of highly uncomfortable feelings reported, but we discussed with each willing participant at the end of the interviews about the training. Moreover, participants were free to abandon the experiment at any moment if they felt uncomfortable.

#### Conclusion of the Interview

##### Control

Participants stated their conclusion regarding the alleged situation and received no feedback (Feedback about the conclusions of the four stories was provided only at the end of the fourth interview).

##### Outcome feedback

Participants stated their conclusion regarding the alleged situation and then received feedback from the researcher regarding what really had happened to the avatar after each interview. Eventual discrepancies between the two versions were highlighted.

##### Process feedback

Participants stated their conclusion regarding the alleged situation and then received feedback on the types of questions they had asked during the interview. The following scheme was used: feedback was given on a total of four different questions after each interview, two times positive feedback on the use of recommended question-types, and two times negative feedback on the use of not recommended questions types. If the participant, later in the interview, continued to commit the same type of error, priority was given to feedback on new types of mistakes.

##### Combination of both types of feedback

Participants received the combination of the procedures used in the previous two experimental groups.

The interviews were videotaped to allow coding of the questions used by the interviewers.

#### Coding of Question-Types and Avatar Responses

The coding of the questions asked by the interviewer was performed by one of the researchers and based on schemes presented in previous research (Lamb et al., 1996, 2000; Sternberg et al., 1997; Korkman, 2006). Descriptions for question-types and details are presented in **Table 1**.

**Table 1:** Description of question-types and details coding used for the experiment.

Category	Definition	Examples
**Recommended questions**		
Facilitators	These questions encourage the child to continue disclosing a certain event without using suggestive words; also requests for clarification were included in this category	“What did you say” “Continue”
Invitations	These questions are open-ended questions that help the child to provide a free recall response, without any suggestive influence by the interviewer. They can be related to the previous statement elicited from the child or related to a new topic.	“Tell me everything about this game” (if the child has already mentioned it),“Tell me all you remember”
Directive	Open-ended and non-suggestive questions that focus child attention on a previously mentioned detail asking for a focalized explanation (usually WH Questions)	“What does bad mean?” “Where did you go with dad?” “Why were you crying?”
**Not-recommended questions**		
Option-posing	These are closed questions that focus the child’s attention on details that the child has not previously mentioned but do not imply a particular type of response, because suggestive techniques are not used. Typical responses to these type of questions are “Yes” “No” or a detail chosen from alternatives provided by the interviewer.	“Do you like him?” “Did she do something bad?” “Who hurts you? Dad or Mom”
Specific suggestive	These are questions in which the interviewer strongly communicates what kind of response is expected using details that the child has never mentioned before	“She touched you, didn’t she?” “I know that someone touched you, tell me who it was!”
Unspecific suggestive	The interviewer strongly communicates what kind of response is expected *avoiding* the use of unmentioned details in these questions. Social pressure and negative feedback to the child’s previous responses belong to this category.	“I know you are a good child so tell me the truth regarding what happened with dad!”
Repetitions	Repeating the question was coded here. These may have a negative feedback effect on children (“My answer was wrong.”) and force them to change their previous answer.	In this category were included all the questions that were repeated more than once
Too-long	Questions must be adapted to the child’s cognitive level. In this category were included all the questions in which more than one concept was present within the same question, or when the interviewer asked several questions in a series	“You stayed more at your father’s house, right? Because he loves you? Which one do you prefer between Mum and Dad?”
Unclear	In this category were included all the questions that contained too difficult words according to the age and the cognitive level of the child and the questions that had been formulated in a haphazard manner	“When you were with your father, this thing that could have happened, it happened also in other occasions?”
**Details**		
Relevant	Details that the avatar utters regarding the alleged CSA event (this type of detail was present in both scenario types)	“Dad touched my willie”
Neutral	Details not linked with the alleged abuse situation but that are related with the avatar’s story and that he or she can remember (this type of detail was present in both scenario types)	“I like to play football”
Wrong	Details related to the alleged abuse situation, that were produced during the interview but that were not present in the avatar’s predefined memories	Interviewer: “Your father asked you to get naked and touch him, didn’t he?” Avatar: “Yes”

## Results

Pearson correlations were used to analyze correlations between the types of questions, the number of details and the conclusions of the interviewer. Because we had a repeated measures design and the residuals of our dependent variables were not normally distributed (Shapiro–Wilk test, *p* < 0.000), we used Generalized-Estimating Equations with independent correlation structure to analyze the effect of process feedback, on details in avatar responses, and on conclusions. We used one-sided tests since we had clear directional hypotheses (e.g., Cho and Abe, 2013).

We decided to run our analyses using the first interview as a covariate while measuring differences between the groups over the three post-feedback interviews. The covariate was included as we found statistical differences between groups during the first interview for the variables relevant details Wald χ^2^(3) = 15.71, *p* = 0.001, and recommended questions Wald χ^2^(3) = 16.48, *p* = 0.001. In some cases, participants in the control group provided the correct conclusion regarding the story when they actually found only one relevant detail, suggesting that they were likely to have guessed when they provided a correct conclusion. Relevant details and conclusions are strictly related since in real life we do not guess regarding the outcome of an alleged abused situation. We decided anyway to keep all the cases in the analyses. We reported effect sizes estimates of the group × time interaction in the pairwise comparison (dppc2) based on the formula for repeated measures designs presented by Morris (2008). These were calculated using the raw means and standard deviations at pretest (first interview) and post-test (average of the last three interviews). The magnitude of dppc2 has been classified as no effect (dppc2 < 0.20), small (dppc2 > 0.20), medium (dppc2 > 0.50), and large (dppc2 > 0.80) following previous suggestions (e.g., de Haan et al., 2015). We also calculated reliable-change indices in order to understand if the participants had significantly changed their questioning style. We used the practice-adjusted reliable change index (RCI), proposed by Chelune et al. (1993), for two main reasons. First, compared to the RCI proposed by Jacobson and Truax (1991), the Chelune formula allowed us to control not only the error of measurement but also the effect of practice, which in a repeated measure design can jeopardize the results. Second, the Chelune formula has been found to perform comparably to more complex regression formulas (Temkin et al., 1999; Frerichs and Tuokko, 2005; Parsons et al., 2009). We used ±1.645 as RCI score to determine if a change was reliable or not (Chelune et al., 1993; Heaton et al., 2001; Collie et al., 2002; Frerichs and Tuokko, 2005; Parsons et al., 2009). We used standard deviations of the whole sample at baseline (the first interview) and we calculated, for the test of the reliability, the single measure intraclass correlation coefficient across all time points from the control group.

Concerning wrong details, we winsorized two outliers because their values were further than three SD from the mean.

### Descriptive Statistics and Correlations of Correctness of Conclusions, Question-Types and Detail Types

Considering all the four interviews, participants reached the correct conclusion in 20% of the cases. The percentage was higher in the group receiving the combination of both types of feedback (29%), and the group receiving process feedback alone (21%); instead, it was lower in the group receiving outcome feedback alone (15%) and in the control group (17%). Participants on average used twice as many not recommended questions (*M* = 26.39, *SD* = 15.65; 65%) per interview compared to recommended questions (*M* = 13.52, *SD* = 7.96; 35%). The most common not recommended question-type used was Option-Posing (*M* = 20.88, *SD* = 13.21) and the least common was Repetition (*M* = 0.65, *SD* = 1.31). The most common recommended question-type used was Directive (*M* = 6.38, *SD* = 6.50) and the least common was Facilitator (*M* = 1.31, *SD* = 2.51). On average, the participants obtained three (*M* = 3.38, *SD* = 2.16) relevant details out of the average maximum of eight details (the maximum varied somewhat from scenario to scenario with a mean of eight), one-and-a-half (*M* = 1.73, *SD* = 1.08) neutral details out of the average maximum of four details and less than one wrong detail (*M* = 0.71, *SD* = 1.35). These results suggest that overall the participants failed to obtain all the available information from the avatars.

### Test of the Algorithms

We expected recommended questions to be positively correlated with relevant and neutral details and negatively correlated with wrong details; we also expected recommended questions to be associated with a higher likelihood of a correct conclusion. At the same time, we expected the reverse for not recommended questions (**Table 2**). As expected, recommended questions were correlated in the expected directions with all the tested variables. The correlation coefficients were statistically significant in all cases, with the exception of wrong details.

**Table 2:** Means, standard deviations, and correlations among questions type details and conclusions.

Variables	*M*	*SD*	1	2	3	4	5	6
1 Total number of recommended	13.52	7.96	–					
2 Total number of not recommended	26.39	15.66	0.16ˆ*	–				
3 Relevant details	3.38	2.16	0.85ˆ**	0.05	–			
4 Neutral details	1.73	1.08	0.93ˆ**	0.12	0.85ˆ**	–		
5 Wrong details	0.63	1.03	0.01	0.42ˆ**	0.04	–0.00	–	
6 Conclusions	0.20	0.40	0.22ˆ**	–0.12	0.27ˆ**	0.24ˆ**	–0.06	–

*^∗∗^*p* < 0.01, ^∗^*p* < 0.05 level.*

Not recommended questions had a positive correlation with wrong details, with a statistically significant coefficient; and a negative correlation with correct conclusions. Contrary to expectations, not recommended questions were positively associated with the number of relevant and neutral details, however, these associations were not statistically significant.

### Effect of Feedback on the Proportion of Recommended and Not Recommended Questions

**FIGURE 1:**
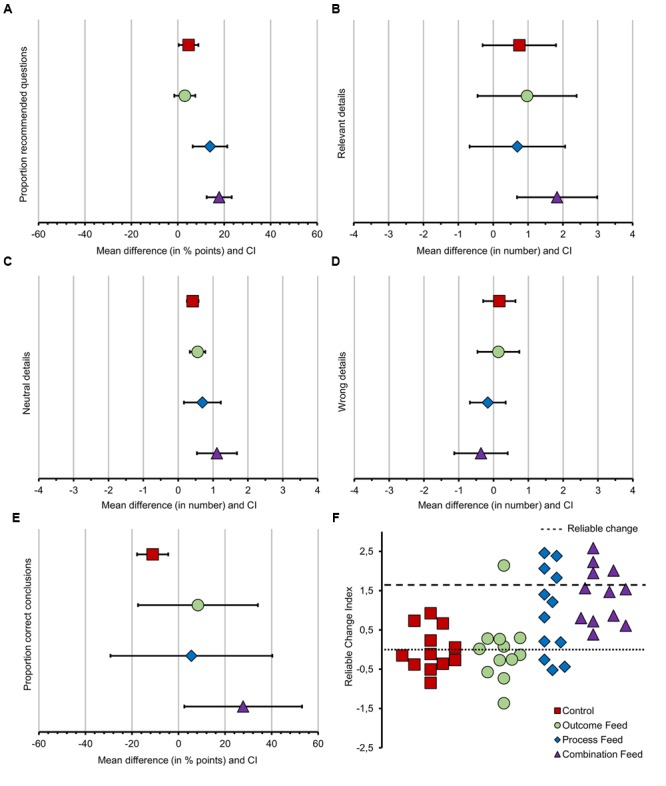
Mean differences between the first interview and the average value of the last three interviews and reliable change by group. In panels **(A–E)** (from left to right), the *x*-axis displays the mean difference with CI between the first and the last three interviews, divided by groups. Panel **(A)** represents the mean gain for the proportion of recommended questions in % points. Panel **(B)** represents the mean gain for the number of relevant details in numbers. Panel **(C)** represents the mean gain for the number of neutral details in numbers. Panel **(D)** represents the mean gain for the number of wrong details in numbers. Panel **(E)** represents the mean gain for the proportion of correct conclusions in % points. Positive numbers reveal a gain while negative numbers reveal a decrease. The only expected decrease was for the number of wrong details. Panel **(F)** displays the reliable change by participant per group.

For the figures, we compared raw observations for means and standard errors during the first interview against the average value over the three last interviews, while the statistical significance was tested in analyses using scores on the same variable from the first interview as covariate. Overall, we found a significant effect of feedback type on the proportion of recommended questions Wald χ^2^(3,144) = 30.63, *p* < 0.001 (**Figure 1A**). Next, we tested our hypotheses with a series of planned comparisons (detailed results of the planned comparison are in **Table 3**).

**Table 3:** Detailed results of the planned comparisons on the dependent variables.

Dependent variable	Comparison	*df*	*Z*	*p*	*d_ppc2_*
% Recommended questions	C vs 3F	1.144	7.44	=0.003	0.53
	OF vs PF	1.72	9.82	=0.001	0.91
	O/PF vs 2F	1.108	8.70	=0.001	0.76
Relevant details	C vs 3F	1.144	13.85	<0.001	0.27
	OF vs PF	1.72	0.80	ns	–0.16
	O/PF vs 2F	1.108	2.58	ns	0.55
Neutral details	C vs 3F	1.144	14.09	<0.001	0.43
	OF vs PF	1.72	1.02	ns	0.15
	O/PF vs 2F	1.108	1.71	ns	0.58
Wrong details	C vs 3F	1.144	3.08	=0.040	–0.22
	OF vs PF	1.72	5.50	=0.009	–0.30
	O/PF vs 2F	1.108	0.89	ns	–0.32
Correct conclusions^1^	C vs 3F	1.144	2.08	ns	0.63
	OF vs PF	1.72	0.35	ns	–0.08
	O/PF vs 2F	1.108	3.17	=0.037	0.64

*C, control group; OF, outcome feedback; PF, process feedback; O/PF, outcome or process feedback; 2F, combination of outcome and process feedback; 3F, all three feedback conditions together.*

*^1^For correct conclusions, effect sizes are calculated on the proportion of correct conclusion.*

#### Control Group vs All Three Feedback Groups

Participants who received any kind of feedback employed a statistically higher proportion of recommended questions (*M* = 41.68, *SE* = 1.56) compared to control group (*M* = 25.93, *SE* = 2.79), which is in line with Hypothesis 1 (**Table 3**).

#### Outcome Feedback vs Process Feedback

Participants who received process feedback employed a statistically higher proportion of recommended questions (*M* = 45.92, *SE* = 2.64) compared to the group who received feedback on conclusions (*M* = 29.90, *SE* = 1.88). This is in line with Hypothesis 2 (**Table 3**).

#### Outcome or Process Feedback vs Combination of Both Types of Feedback

Participants who received a combination of both types of feedback used a statistically higher proportion of recommended questions (*M* = 49.22, *SE* = 2.42) compared to participants who received only one of the two types of feedback (*M* = 37.90, *SE* = 1.87). This is in line with Hypothesis 3 (**Table 3**).

### Effect of Feedback on Details Elicited from the Avatars

Overall, we found a significant effect of feedback type on the total number of relevant details Wald χ^2^(3,144) = 22.17, *p* < 0.001, neutral details Wald χ^2^(3,144) = 21.03, *p* < 0.000, and wrong details Wald χ^2^(3,144) = 11.47, *p* = 0.004 (see **Figures 1B–D** and **Table 3**).

#### Control Group vs All Three Feedback Groups

Participants who received any kind of feedback elicited on average more relevant details (*M* = 4.17, *SE* = 0.20) compared to control group (*M* = 2.08, *SE* = 0.28); more neutral details (respectively *M* = 2.18, *SE* = 0.10 vs *M* = 1.08, *SE* = 0.13), and fewer wrong details (respectively *M* = 0.56, *SE* = 0.10 vs *M* = 0.92, *SE* = 0.19). These results are in line with Hypothesis 1 (**Table 3**).

#### Outcome Feedback vs Process Feedback

Participants who received process feedback elicited on average more relevant details (*M* = 4.36, *SE* = 0.34) compared to the group who received feedback on conclusions (*M* = 3.56, *SE* = 0.36) and more neutral details (*M* = 2.28, *SE* = 0.17 vs *M* = 1.89, *SE* = 0.17). However, these differences were not significant. Moreover, they elicited fewer wrong details (*M* = 0.25, *SE* = 0.11 vs *M* = 0.97, *SE* = 0.21). The last difference was statistically significant. Thus in line with Hypothesis 2 (**Table 3**).

#### Outcome or Process Feedback vs Combination of Both Types of Feedback

Participants who received a combination of both types of feedback elicited on average more relevant details (*M* = 4.58, *SE* = 0.34) compared to participants who received only one of the two types of feedback (*M* = 3.96, *SE* = 0.25); more neutral details (respectively *M* = 2.36, *SE* = 0.16 vs *M* = 2.08, *SE* = 0.12); and fewer wrong details (respectively *M* = 0.47, *SE* = 0.15 vs *M* = 0.61, *SE* = 0.13). None of these differences was significant. These results were not in line with Hypothesis 3 (**Table 3**).

### Effect of Feedback on the Correctness of the Conclusions of the Participants

Overall, we found no significant effect of feedback type on the proportion of correct conclusions. Results are shown in **Figure 1E**.

#### Control Group vs All Three Feedback Groups

Participants who received any kind of feedback drew on average more correct conclusions (*M* = 0.25, *SE* = 0.04 vs *M* = 0.14, *SE* = 0.06). However, the difference was not significant. This result was not in line with Hypothesis 1 (**Table 3**).

#### Outcome Feedback vs Process Feedback

Participants who received process feedback drew on average more correct conclusions (*M* = 0.22, *SE* = 0.07 vs *M* = 0.17, *SE* = 0.06). Also here, the difference was not significant. This result was not in line with Hypothesis 2 (**Table 3**).

#### Outcome or Process Feedback vs Combination of Both Types of Feedback

Participants who received a combination of both types of feedback drew on average significantly more correct conclusions (*M* = 0.36, *SE* = 0.08 vs *M* = 0.19, *SE* = 0.05). This result was statistically significant and was in line with Hypothesis 3 (**Table 3**).

### Reliable Change on Proportion of Recommended Questions

Nine participants were able to reach a reliable change in the proportion of recommended questions used during the interviews, one from the outcome feedback group and four from both question type feedback and combination feedback groups. Remarkably, only participants in the combination feedback group were always able to improve their performance. Results are shown in **Figure 1F**.

## Discussion

The research reported in the current study had two aims. First, it replicated previous findings regarding the effect of training and feedback in interviews with avatars in alleged sexual abuse scenarios. Second, it provided further information regarding the characteristics of effective feedback. Thanks to the structure of our simulated interview using avatars with prerecorded memories, we were able to provide an immediate, detailed and unbiased outcome feedback to our participants in addition to feedback on the questions used during the interviews. This is almost never possible in real CSA investigations because the ground truth of a case is rarely known.

### Effects of Different Types of Feedback

We tested the effect of three types of feedback taken together compared to the control group. From a multivariate perspective, considering the pattern of effects across several outcome measures, feedback was shown to improve the quality of investigate interviews. Effect size estimates suggested small to medium effects.

However, in this study we also wanted to compare the effects of different type of feedback. This study confirmed previous findings in other simulation training based environments (e.g., Astwood et al., 2008), the planned comparisons showed a general superior effect of process feedback over outcome feedback. From a multivariate perspective, the results where somewhat mixed in this comparison. A strong effect was present for the proportion of recommended questions, which led to a moderate effect in the number of wrong details elicited, but no effects were found for the other variables of interest. This can be explained by the fact that process feedback substantially improved participant performance in the task they have been trained of (process feedback was provided only on the question-types used) but these improvements did not lead to substantial differences on the other variables. It is important to highlight that this can be related to the few interviews performed. Outcome feedback instead showed the expected weakest effects. A possible explanation is that outcome feedback, compared to process feedback, does not provide any guidance for the interviewer on how to perform better. Moreover, consistent literature shows how novice learn more deeply with process feedback than outcome feedback (e.g., Mayer and Johnson, 2010).

The most important finding of this study is the effect related to the simultaneous combination of the two types of feedback. The combination of feedback, showed the strongest effect in improving the proportion of recommend questions used by the interviewer and the proportion of correct conclusions. A possible explanation of these results is that the combination of both types of feedback provided the participants with both a reason and a direction for change. The mean difference, between the baseline and the average level of the last three interviews, clearly shows that the combination feedback led to bigger improvements in all the variables compared to the other two feedback types and compared to the control group. This is indeed an important result because it shows that the combination of feedback has a stronger general effect on interview quality. An interviewer aiming not to jeopardize the child account should maximize not only the proportion of recommended questions, but also improve the number of correct details and limit the number of wrong details. As consequence of these improvements, also the probability to provide a correct conclusion is positively influenced.

The previous results are also confirmed by the analyses concerning reliable change; on an individual level only the combination of both feedback improved the proportion of recommended questions of every participant.

It is important to highlight that our evaluation of correct conclusion was strict, which may and explains why the overall effects are small. In order to achieve a correct conclusion all the details regarding the story had to be correct and the participants have only 10 min to gather all the information. In summary, in the light of small number of interviews provided and the rigorous experimental method used, these results are remarkable.

### Limitations

The sample used in this study was relatively small thereafter is not possible to conclude definitive results. Moreover, because we delivered only four interviews, it is possible that the training was quite short and that longer sessions would increase the feedback effect. Finally, no second evaluation of the coding has been done. An important aspect that also remains unexplored is the longevity of these effects over time.

## Conclusion and Future Directions

In the current study, the combination of outcome and process feedback showed the stronger effect on interviews quality compared to the two type of feedback taken separately. To our best knowledge, this is the first study to investigate the two types of feedback, separately and combined, in investigative interviews in CSA cases. The current study also replicated the overall positive effects of feedback and training with avatar on the quality of investigative interviews. Based on the outcomes of the current study, we believe that one of the strengths of simulated investigative interviews using avatars in this context is the possibility to provide an immediate, continuous, and detailed feedback in a cheap (both monetary and time-wise) way. This is an important feature since researchers have not yet identified any less costly techniques than providing practice and supervision. The next step is to replicate these results with a bigger sample of professional interviewers. A possible future direction is the inclusion of the avatar interview session as part of the students’ placement unit.

## Ethics Statement

This study was carried out in accordance with the recommendations of Ethics Board of the Department of Psychology and Logopedics at Åbo Akademi, with written informed consent from all subjects. All subjects gave written informed consent in accordance with the Declaration of Helsinki. The protocol was approved by the Ethics Board of the Department of Psychology and Logopedics at Åbo Akademi.

## Author Contributions

All authors listed have made substantial, direct and intellectual contribution to the work, and approved it for publication. More specifically, FP, AZ, and PS designed the study; FP performed the experiment, FP and PS analyzed and interpreted the data, and FP drafted the manuscript. JA, AZ, and PS critically reviewed the manuscript.

## Conflict of Interest Statement

The authors declare that the research was conducted in the absence of any commercial or financial relationships that could be construed as a potential conflict of interest.

## Abbreviations

CSA: child sexual abuse
EIT: Empowering Interviewer Training
